# Folic Acid Attenuates *N*-Methyl-*N*’-Nitro-*N*-Nitrosoguanidine-Induced Gastric Mucosal Injury in Rats

**DOI:** 10.5152/tjg.2024.23506

**Published:** 2024-11-01

**Authors:** Caiting Peng, Li Wang, Yuan Liang, Li Che, Rongjing Sun, Jia Yu, Jiamin Gong, Dandan Wang, Suizhi Cheng, Qingqing Yang, Tao Jing, Zhenzhong Liu

**Affiliations:** 1 North Sichuan Medical College, Nanchong, China; 2 Huazhong University of Science and Technology, Wuhan, Hubei, China

**Keywords:** Chemoprevention, folic acid, *N*-methyl-*N*’-nitro-*N*-nitrosoguanidine, NF-κB/NLRP3, stomach

## Abstract

**Background/Aims:**

*N*-Methyl-*N*’-nitroso-*N*-nitrosoguanidine (MNNG) is suspected to increase the risk of developing stomach cancer. Folic acid (FA) is familiar with decreasing inflammation. We expected that FA would protect against MNNG-induced gastric mucosal injury.

**Materials and Methods:**

Thirty 12-week-old SPF-grade female Sprague-Dawley (SD) rats were treated with MNNG and given different dosages of FA as an intervention measure. Quantitative polymerase chain reaction (qPCR) was used to analyze the expression of IL-1, IL-6, IL-8, IL-18, TNF-α, NLRP3, ASC, and caspase-1 genes. The enzyme-linked immunosorbent assay (ELISA) was utilized for the identification of inflammatory cytokines. Western blot was accustomed to detecting IL-1β, IL-18, and NLRP3 inflammatory vesicles in gastric tissue. Furthermore, the gastric mucosal tissues underwent histological examination.

**Results:**

Our investigation demonstrated that FA reduced MNNG-induced inflammatory factor increase by decreasing NF-κB signaling (*P *< .05). Furthermore, FA prevented the MNNG-induced upregulation of NLRP3 inflammasome-related genes and proteins (all *P* < .01).

**Conclusion:**

Our data imply that MNNG exposure stimulates the NF-κB/NLRP3 pathway, while FA suppresses it, limiting stomach mucosal inflammation.

**Main Points**

- MNNG induced increased expression of NLRP3 inflammasome-associated mRNAs and proteins.
- MNNG treatment induced elevated expression of NLRP3, ASC, and caspase-1.
- FA alleviated the MNNG-induced inflammatory response by inhibiting NLRP3 inflammasome.

## Introduction

Gastric cancer ranks fifth among common malignant tumors worldwide.^1^ Gastritis often leads to gastric mucosal lesions, which can progress to cellular abnormalities and eventually develop into cancer.^2,3^ Several factors influence the occurrence of GC, with notable emphasis on an imbalanced diet, excessive consumption of preserved foods, and inadequate intake of vegetables and fruits.^4-7^

N-nitroso compounds (NOCs), particularly *N*-methyl-*N*’-nitroso-*N*-nitrosoguanidine (MNNG), are potent carcinogens that have been identified as key contributors to gastric cancer.^8-10^ *N*-methyl-*N*’-nitroso-*N*-nitrosoguanidine is a chemical commonly used to construct an animal model of gastric mucosal injury.^11-14^ Conversely, epidemiological studies have shown that consuming fresh vegetables rich in FA can lower the chances of GC to some extent and alleviate inflammation-induced damage in the esophagus and stomach caused by MNNG.^16,17^ Many in vitro and animal studies have suggested that FA deficiency may contribute to carcinogenic events. Low FA intake was associated with increased expression of immune-related genes, urokinase, and iNOS, and downregulation of genes encoding the adhesion protein procadherin-4. Epidemiologic and clinical studies have shown that both FA intake and blood levels are inversely associated with colorectal cancer risk. Similarly, FA intake was positively associated with the risk of recurrence in patients with non-muscle invasive bladder cancer.^18-19^

At the cellular biology level, NF-κB typically exerts its effects as a p50/p65 complex, and IKK-mediated IκB phosphorylation is a critical mechanism for NF-κB nuclear translocation.^20-23^ Additionally, the NLRP3 inflammasome, composed of NLRP3, ASC, and pro-caspase-1, plays a vital role in inflammatory regulation.^24,25^

Against this backdrop, our study employed an MNNG-induced rat model of gastric mucosal injury and introduced FA to mitigate MNNG-induced damage. Moreover, we investigated whether FA could protect the gastric mucosa by inhibiting the NF-κB/NLRP3 pathway. This research enhances our understanding of the toxic pathways of MNNG. Concurrently, it presents a fresh perspective on the significance of FA in protecting gastric mucosa.

## Materials and Methods

### Animal Experiments

Animal models were established based on previous reports.^26^ Specifically, a total of 30 female 8-week-old SD rats weighing 180-220 g were obtained from the North Sichuan Medical College Laboratory Animal Center. SPF rats were kept in typical conditions with a 12-hour light/darkness cycle. The temperature and humidity of the rats’ environment were carefully controlled.

In the early stage of the experiment, we reviewed a large number of literature, combined with the previous relevant research of our research group, designed and conducted a pre-experiment, and adjusted the experimental doses of MNNG and FA according to the results of the pre-experiment. The rats were divided into 5 groups through random selection: control group, MNNG group (administered 25 mg/kg MNNG), low-dose FA group (given 25 mg/kg MNNG + 1.25 mg/kg FA), medium-dose FA group (administered 25 mg/kg MNNG + 2.5 mg/kg FA), and high-dose FA group (received 25 mg/kg MNNG + 5 mg/kg FA).

In the initial week of grouping, the FA groups’ rats were given the appropriate FA doses for a duration of 12 hours, with normal saline being given for the subsequent 12 hours. The control and MNNG groups received no additional treatment and were fed normally. From the second week onward, the FA groups continued to receive FA for 12 hours, followed by the administration of 25 mg/kg MNNG for the next 12 hours. Normal saline with a final concentration of 0.2% DMSO was administered to the control group for 24 hours. MNNG was dissolved in DMSO with a consistent final concentration of 0.2% across all groups.

The MNNG poisoning animal models were established through oral administration. Gastric tissues were washed with normal saline for pathological observation. Serum from centrifuged blood samples and gastric mucosal tissues were reserved at −80°C until further use. The study was confirmed by the North Sichuan Medical College Ethics Committee (approval no: 02/2022,date: February 7, 2021), and all procedures were conducted following the guidelines of the National Institutes of Health guide of the care and use of laboratory animals (NIH Publications No. 8023, revised 1978).

### Real-Time Quantitative PCR

The control group was given normal saline with a 0.2% DMSO concentration for a total of 24 hours. Specific primers for amplification were integrated by Wuhan Tianyi Huayu Gene Technology Co., Ltd. Table 1 shows the primer sequences. Experiments were conducted using GoTaq qPCR Master Mix (Promega, Madison, Wisconsin, America). All PCR reactions were performed on BIO-RAD CFX96 Real-Time System (BIO-RAD, Hercules, California, America) with 3 replicates (n = 3). The levels of gene expression for the target genes were normalized to the expression of the housekeeping gene β-actin, and the relative quantity of mRNA was determined using the 2^-ΔΔCt^ method.

### Enzyme-Linked Immunosorbnent Assay

Enzyme-linked immunosorbnent assay kits from RUIXIN BIOTECH (Quanzhou, China) were used for this purpose. The specific ELISA kits used were as follows: Rat IL-1β ELISA Kit (Cat.# RX302869R;), Rat IL-6 ELISA Kit (Cat.# RX302856R), Rat IL-8 ELISA Kit (Cat.# RX302854R), Rat IL-18 ELISA Kit (Cat.# RX302871R), and Rat TNF-α ELISA Kit (Cat.# RX302058R). The absorbance of the sample was measured using an ELISA reader at 450 nm (Bio-Rad, Hercules, CA, America). The obtained data were then analyzed and the results were calculated.

### Western Blotting

Concisely, proteins were treated with RIPA lysis buffer (Beyotime, Shanghai, China). Next, the protein content was detected by a BCA protein assay kit (Beyotime, Shanghai, China). Moreover, they were separated by SDS-PAGE and transferred onto a polyvinylidene difluoride membrane. Following incubation in TBST containing 5% bovine serum for 2 hours, the membranes underwent an overnight incubation with the designated antibodies: IKKα antibody (ab30241; abcam), p-IKKα antibody (2697; Cell Signaling Technology), IκBα antibody (10268-1-AP; proteintech), p-IκBα antibody (ab133462; abcam), p65 antibody (BA0610; BOSTER), p-p65 antibody (ab76302; abcam), NLRP3 antibody (ab263899; abcam), ASC antibody (A1170; ABclona), pro caspase-1 antibody (ab179515; abcam), caspase-1 p10 antibody (AF4022; Affinity), PTEN antibody (22034-1-AP; proteintech), PI3K antibody (AF5112; Affinity), AKT antibody (60203-2-lg; proteintech), p-AKT antibody (28731-1-AP; proteintech), and mTOR antibody (66888-1-lg; proteintech). Following the overnight incubation with primary antibodies, the membranes were incubated with secondary antibodies: HRP Labeled Goat Anti-rabbit IgG (BOSTER, Wuhan, China) and HRP Conjugated AffiniPure Goat Anti-mouse IgG (H+L) (BOSTER, Wuhan, China). Results were detected and visualized utilizing an enhanced chemiluminescence system and the Integrated Chemiluminescence Imager Chemiscope S6 (Qinxiang Scientific Instruments Co., Ltd., Shanghai, China). The ImageJ software was utilized for quantifying the polypeptide bands, with β-actin serving as the internal reference.

### Hematoxylin and Eosin Staining

The samples underwent dehydration using a fully automatic dehydrator and were then wrapped in paraffin. Sections were obtained from the implanted tissues and subjected to staining. The tissue sections were visualized using the Pannoramic 250 digital scanner (3DHISTECH, Budapest, Hungary), which captured images of the slices.

### Immunohistochemical Study

The deparaffinization and dehydration of sections were accomplished using xylene and ethanol solutions. Citrate buffer was utilized for antigen retrieval, with a 20-minute treatment in a microwave oven. Following PBS rinsing, 3% hydrogen peroxide was applied to cells to block endogenous peroxidase activity. They were stained with DAB substrate and then counterstained using hematoxylin. Subsequently, they were imaged using an Olympus microscope (Olympus), and the resulting images were analyzed with Image-Pro Plus 6.0 software (Media Cybernetics).

### Statistical Analysis

The experimental data were analyzed using Statistical Product and Service Solutions version 20 (IBM SPSS Corp.; Armonk, NY, USA), GraphPad Prism version 8.0 (GraphPad Software; California, America), and all experiments were repeated 3 times. The results were presented as the mean value ± SD. Data were analyzed using one-way analysis of variance by LSD pairwise comparison. *P *< .05 was regarded as statistically significant.

## Results

### Effects of MNNG Exposure and FA Intervention on IL-6, IL-8, TNF-α, and NF-κB in the Gastric Mucosa of Rats

TNF acted as proximal mediators and induced production of other mediators including IL-6 and IL-8. In humans, TNF-α, IL-6, and IL-8 are regulated to some degree by NF-κB activation. NF-κB is a ubiquitous protein transcription factor that enhances the transcription of a variety of genes. NF-κB normally resides in the cytoplasm, where it is retained by association with the IκB protein, an endogenous inhibitor of NF-κB. However, when activated, it translo cates to the nucleus, binds the DNA, and activates genes. The activation involves the phosphorylation, ubiquitination, and degradation of IκB, leading to the nuclear migration of NF-κB.

**Figure 1.**
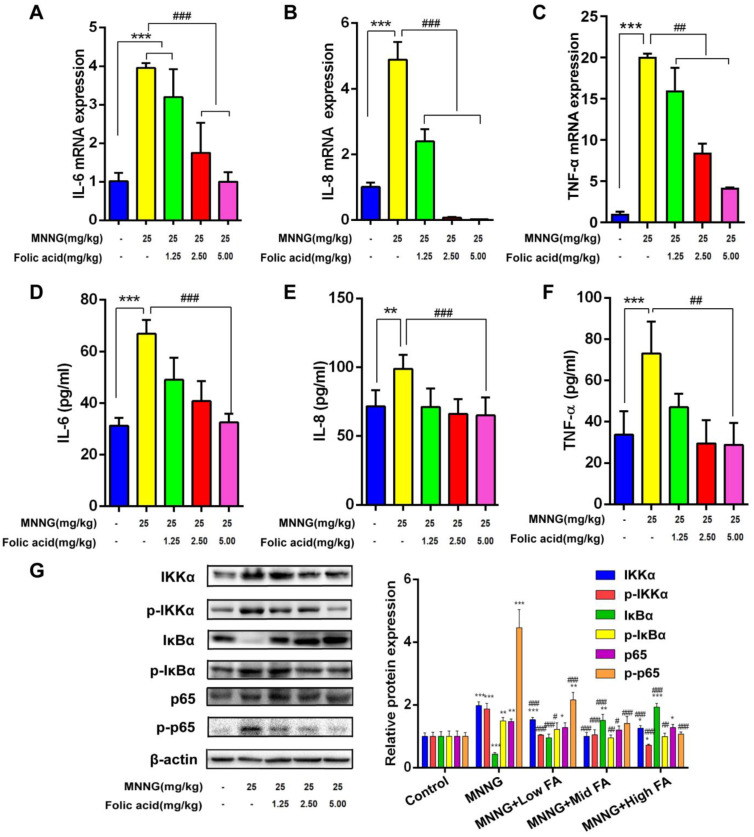
Effects of MNNG exposure and FA intervention on inflammatory response in the gastric mucosa of rats. (A, B, and C) represented the mRNA expression of IL-6, IL-8, and TNF-α, respectively, in different rat groups. (D, E, and F) represented the contents of IL-6, IL-8, and TNF-α, respectively, in different rat groups. (G) represented the levels of NF-κB signaling-related proteins after MNNG exposure and FA intervention. n=3, compared with the control group, *P<0.05, **P<0.01, ***P<0.001; compared with the MNNG group, #*P*<0.05, ##*P*<0.01, ###*P*<0.001.

The experimental findings revealed that FA effectively mitigated the up-regulation of the above-mentioned mRNA expression induced by MNNG (Figure. 1A-C). Especially, as the dosage of FA intervention increased, the mRNA expression levels of these inflammatory markers gradually lessened. Next, we used an ELISA assay to examine the impact of FA on the expression of the above-mentioned factors induced by MNNG. The results demonstrated that FA successfully alleviated the MNNG-induced elevation in the expression of the above-mentioned factors(Figure. 1D-F). Besides, a decrease in the levels of inflammatory markers was noted with an increase in the dosage of FA intervention.

Next, we investigated the protein levels associated with NF-κB signaling. Upon exposure to MNNG, we observed an increase in the levels of IKKα, p-IKKα, p-IκBα, p65, and p-p65, accompanied by a decrease in IκBα levels (Figure. 1G). However, following intervention with different doses of FA, notable changes in these protein levels were observed. Specifically, the protein levels of IKKα, p-IKKα, p-IκBα, p65, and p-p65 decreased, while the IκBα protein level increased (all *P *< .05, Figure 1G).

### Histopathological Changes in Gastric Tissues of Rats After Pharmacological Intervention

**Figure 2.**
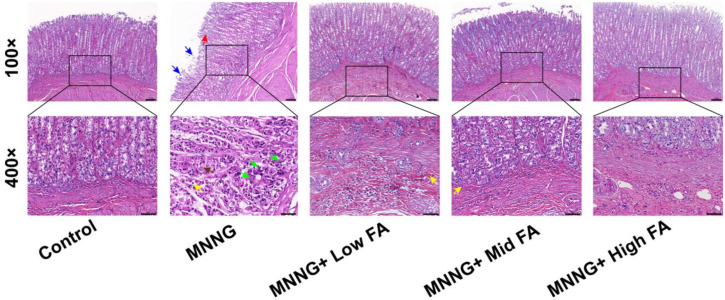
Histopathological changes in gastric tissues of rats after MNNG exposure and FA intervention. The yellow arrow represents neutrophils infiltration, the red arrow represents the hyperplasia of gastric gland cells, the brown arrow represents gastric gland cells degeneration, the blue arrow represents sloughing of gastric mucosal epithelium, and the green arrow represents cell necrosis and disintegration (magnification ×100 and scale bar 200 μm; magnification ×400 and scale bar 100 μm).

Upon histological examination, MNNG exposure caused significant injury. The injury manifested as the presence of lymphocytes and neutrophils in the lamina propria, submucosa, and serosa. Additionally, hyperplasia of gastric gland cells was observed in the superficial mucosa. *N*-methyl-*N*’-nitroso-*N*-nitrosoguanidine exposure also led to vacuolar degeneration and necrosis of gastric parietal cells, as well as uneven cytoplasmic staining and nuclear pyknosis or fragmentation. Furthermore, the base of the gastric glands showed local cell necrosis with a blurred structure. Disordered arrangement of gastric glands and pronounced cell hyperplasia were evident in specific mucosal areas, accompanied by a decrease in cytoplasmic basophilia. In contrast, the low-dose group treated with FA displayed infiltration of inflammatory cells, primarily neutrophils, in the submucosa. However, the pathological changes in the anterior stomach were not obvious. In the medium and high doses of FA intervention groups, a few inflammatory cells, primarily lymphocytes and eosinophils, were distributed throughout the lamina propria (Figure 2).

### Effects of MNNG Exposure and FA Intervention on IL-1β, IL-18, and NLRP3 Inflammasome in the Gastric Mucosa of Rats

Several molecular and cellular events have been proposed as the trigger(s) for NLRP3 inflammasome activation, including K efflux, Ca^+2+^ signaling, reactive oxygen species (ROS), mitochondrial dysfunction, and lysosomal rupture. Expression of NLRP3 is induced by priming with microbial components such as TLR ligands or endogenous molecules, such as tumor necrosis factor and IL-1β, through the activation of NF-κB. The NLRP3 inflammasome controls the activation of caspase-1 and the release of the pro-inflammatory cytokines IL-1β and IL-18 in macrophages.

**Figure 3.**
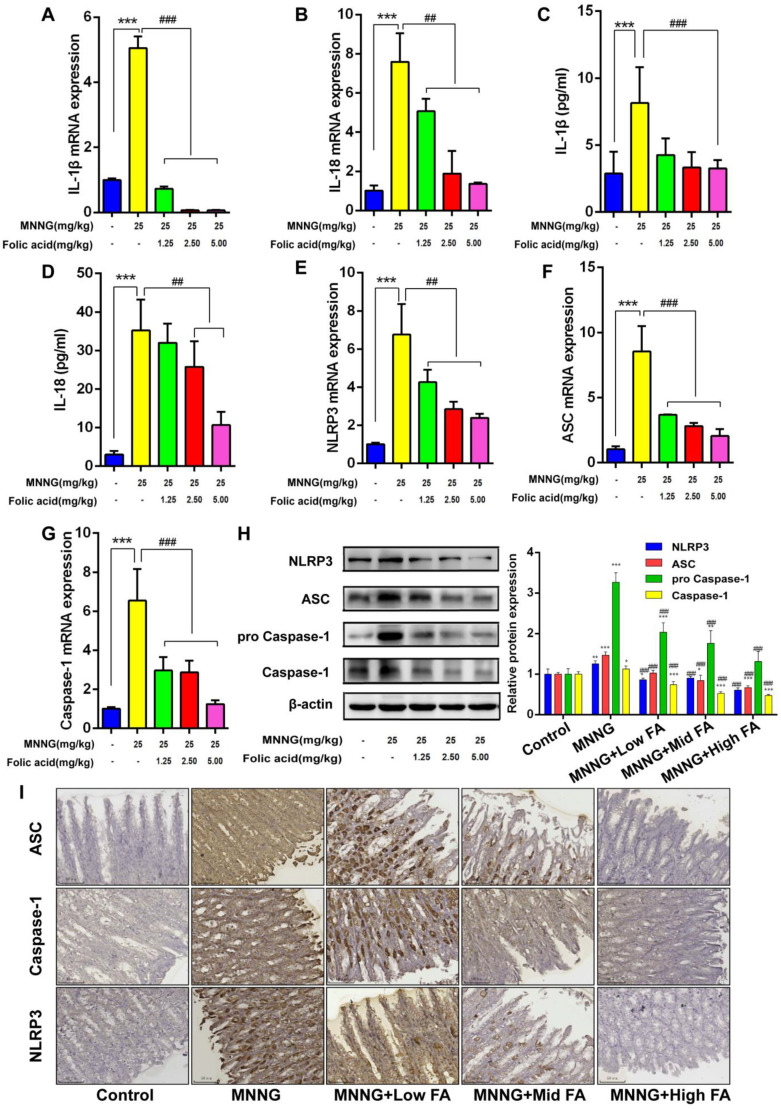
**Effects of MNNG exposure and FA intervention on NLRP3 inflammasome-related genes and proteins in the gastric mucosa of rats.** (A and C) represented the IL-1β and IL-18 mRNA expression, respectively, in different rat groups. (B and D) represented the IL-1β and IL-18 contents, respectively, in different rat groups. (E, F, and G) represented the NLRP3, ASC, and Caspase-1 mRNA expression, respectively, in different rat groups. (H) represented the NLRP3, ASC, pro Caspase-1, and Caspase-1 protein levels after MNNG exposure and FA intervention. (I) Immunohistochemical staining was used to detect the protein expression of ASC, Caspase-1 and NLRP3 in each group. The staining of ASC, Caspase-1 and NLRP3 was brown yellow (50μm scale bar). n=3, compared with the control group, *P<0.05, **P<0.01, ***P<0.001; compared with the MNNG group, #*P*<0.05, ##*P*<0.01, ###*P*<0.001.

In order to assess the impact of FA intervention on inflammation-related gene and protein expression in the gastric mucosa, mRNA and protein levels were measured. Results demonstrated that different doses of FA intervention effectively mitigated the notable increase in IL-1β and IL-18 expression (all *P* < .01, Figure 3A-D). Notably, the high-dose FA group displayed a more pronounced result in reducing the expression levels. Subsequently, the expression of NLRP3 inflammasome-related substances was detected. The findings revealed that different doses of FA intervention attenuated the significant elevation of the above-mentioned factors’ mRNA and protein expression caused by MNNG (all *P* < .01, Figure 3E-H).

Following MNNG intervention, there was a notable increase in ASC, NLRP3, and caspase-1 expression as demonstrated by immunohistochemical analysis. The immunopositive area of FA dose groups was obviously reduced compared to that of the model group. This suggests that FA can reduce the expression of the above-mentioned factors (Figure 3I). Moreover, it was observed that the effectiveness of the intervention increased with higher doses of FA.

**Effects of MNNG exposure and FA intervention on the PI3K/AKT pathway in gastric mucosa of rats**

**Figure 4.**
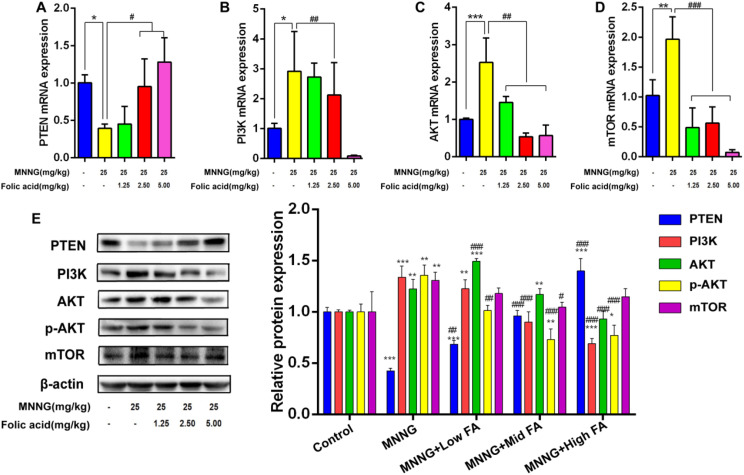
Effects of MNNG exposure and FA intervention on the PI3K/AKT pathway in gastric mucosa of rats. (A-D) The mRNA expression of PTEN, PI3K, AKT, and mTOR after MNNG exposure and FA intervention. (E) The levels of PTEN, PI3K, AKT, p-AKT, and mTOR proteins after MNNG exposure and FA intervention. n=3, compared with the control group, *P<0.05, **P<0.01, ***P<0.001; compared with the MNNG group, #*P*<0.05, ##*P*<0.01, ###*P*<0.001.

In this study, we detected the levels of key genes and proteins of the PI3K/AKT pathway associated with tumorigenesis. The expression of PTEN mRNA was found to be decreased in the MNNG group (*P*<0.05), while PTEN mRNA presented an uptrend with the treatment of FA at different doses (Figure 4A). The mRNA expression of PI3K, AKT, and mTOR was enhanced after the administration of MNNG, whereas FA reduced the PI3K, AKT, and mTOR mRNA (Figure 4B-D).

And then the levels of PTEN, PI3K, AKT, p-AKT, and mTOR proteins were determined via western blot analysis. We found that the MNNG group revealed a remarkable reduction in the PTEN protein level (*P*<0.001), while FA intervention displayed an increase in the level of PTEN as the FA doses escalated (*P*<0.01 or *P*<0.001). In addition, in comparison with the control group, the levels of PI3K, AKT, p-AKT, and mTOR proteins were up-regulated in the MNNG group (*P*<0.01 or *P*<0.001), which were down-regulated in the FA high-dose group compared to the MNNG group (Figure 4E). Taking these results into consideration, it can be concluded that exposure to MNNG can activate the PI3K/AKT signaling pathway, which can be ameliorated by FA. 

## Discussion

Chemical carcinogens, especially MNNG, are central to mimicking NOC, leading to the malignant transformation of normal gastric mucosal cells.^27-30^ Although FA has been shown to inhibit gastric mucosal inflammation, its specific mechanism of action needs to be studied.^31-33^ According to our research, FA successfully shielded SD rats’ stomach mucosa against harm caused by MNNG. The underlying mechanism may be related to the NF-κB/NLRP3 signaling pathway regulation.

In regulating inflammatory and immune responses, cytokines play an indispensable role.^34^ Prolonged exogenous stimulation of the gastric mucosa leads to continuous activation of local inflammation, characterized by an increase in pro-inflammatory cytokines. This disruption of tissue homeostasis exacerbates gastric mucosal damage and may even lead to gastric cancer.^35,36^ Our study demonstrated that FA treatment effectively inhibited the MNNG-induced elevation of pro-inflammatory cytokines, consistent with previous findings.^37^ Additionally, FA attenuated MNNG-induced pathological changes, including inflammatory cell infiltration, disorders in gastric glandular arrangement, and abnormal cell proliferation, indicating its positive role in suppressing gastric mucosal inflammation.

To delve into the specific mechanisms of FA resistance to inflammation, we tested the changes in the NF-κB/NLRP3 signaling pathway. Normally, NF-κB is not active because it binds to the inhibitory protein IκBα.^38^ However, MNNG-induced gastric mucosal inflammation activates NF-κB and its downstream NLRP3 inflammasome, resulting in aggravated inflammatory injury.^39^ Our research demonstrated that treatment with FA notably reduced the NF-κB pathway activation induced by MNNG.^18^

NLRP3, as a sensor molecule of the inflammasome, contributes significantly to the inflammatory response.^40^ The NF-κB pathway is involved in the initiation of the NLRP3 inflammasome.^41,42^ Our study illustrated the inhibitory effect of FA on the activation of the NLRP3 inflammasome induced by MNNG, a process primarily governed by NF-κB. We found that FA exerted a protective effect by inhibiting the aforementioned pathway.

The NLRP3 inflammasome has contributed more to regulating liver inflammation and is closely related to the progression of inflammation in colitis models.^43-46^ Our study not only offers a theoretical basis for the way of protecting the gastric mucosa but also provides a new direction for understanding its role in various diseases. However, one of the limitations of this study is that it did not consider the fact that FA may also be involved in the prevention of gastric cancer through other relevant pathways. At the same time, the interaction between various pathways has not been elucidated.

In summary, this study brings brilliant ideas and strategies for the prevention and treatment of gastric cancer by investigating the inhibitory effect of FA on MNNG-induced gastric mucosal inflammation and its basal mechanism. In the end, we will probe the specific effect of FA in protecting against gastric cancer and its interaction with other signaling pathways. Our goal is to offer improved methods for preventing and treating gastric cancer.

**Figure 5.**
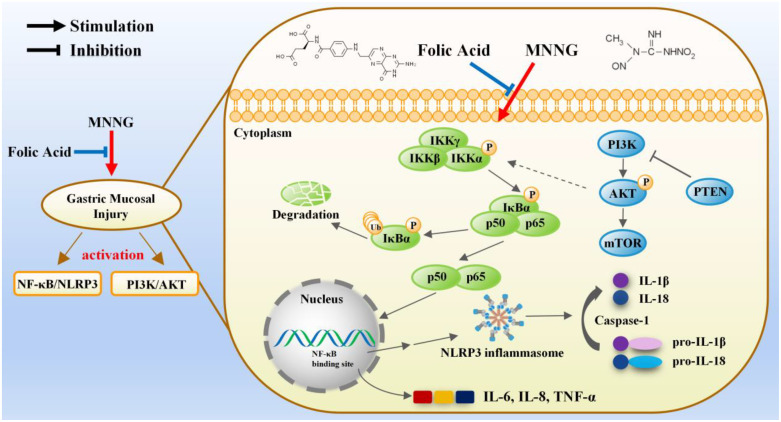
Schematic diagram illustrating the toxicity mechanism of MNNG in the gastric mucosa and the protection mechanism of FA against MNNG-induced gastric mucosal injury. Upon exposure to MNNG, the NF-κB signaling pathway is effectively activated in the gastric mucosa of rats. This induces the transcription of NLRP3, as well as pro-inflammatory cytokines such as IL-6, IL-8, and TNF-α. Formation of the NLRP3 inflammasome and subsequent auto-catalysis of pro Caspase-1 lead to the proteolytic cleavage of pro-IL-1β and pro-IL-18, resulting in the production of mature IL-1β and IL-18, respectively, thereby exacerbating the inflammatory response. Additionally, MNNG exposure activated the PI3K/AKT pathway to augment gastric mucosal injury. Importantly, FA provides enhanced protection against MNNG-induced gastric mucosal injury by inhibiting the NF-κB/NLRP3 and PI3K/AKT pathways.

Taken together, our research suggests that FA diminishes MNNG-induced damage to the gastric mucosa, and the possible explanation lies in the modulation of the NF-κB/NLRP3 signaling pathways. Importantly, we investigated the association between MNNG and the NLRP3 inflammasome, as evidenced by the elevation in NLRP3, ASC, and caspase-1 mRNA and protein levels following MNNG administration. Furthermore, the anti-inflammatory activity of FA contributes to gastroprotection by inhibiting NLRP3 inflammasome activation. Our study reveals new perspectives on the toxicity mechanism of MNNG and provides effective interventions for NOC-induced gastric mucosal injury (Figure 5). In the future, we plan to conduct in-depth screening of other pathways of FA in the prevention of gastric cancer and further study the relationship between each pathway to supplement and improve the systematic research on the prevention and treatment of gastric cancer.

## Figures and Tables

**Table 1.** List of Primers Used in the Study

Gene	Forward Primer (5’-3’)	Reverse Primer (5’-3’)
β-actin	CACTATCGGCAATGAGCGGTTCC	ACTGTGTTGGCATAGAGGTCTTTACG
IL-1β	AGGTCGTCATCATCCCACGAG	GCTGTGGCAGCTACCTATGTCTTG
IL-6	ACTTCCAGCCAGTTGCCTTCTTG	TGGTCTGTTGTGGGTGGTATCCTC
IL-8	CATTAATATTTAACGATGTGGATGCG	GCCTACCATCTTTAAACTGCACAAT
IL-18	CGCAGTAATACGGAGCATAAATGAC	GGTAGACATCCTTCCATCCTTCAC
TNF-α	GAGAGATTGGCTGCTGGAAC	TGGAGACCATGATGACCGTA
NLRP3	TGCATGCCGTATCTGGTTGT	ATGTCCTGAGCCATGGAAGC
ASC	ACAGTACCAGGCAGTTCGTG	GGTCTGTCACCAAGTAGGGC
Caspase-1	GACCGAGTGGTTCCCTCAAG	GACGTGTACGAGTGGGTGTT

## Data Availability

The data that support the findings of this study are available on request from the corresponding author.
